# Differential effects of task difficulty on target-type switching in haptic foraging: Evidence for increased switching with extreme task demands

**DOI:** 10.3758/s13414-025-03064-z

**Published:** 2025-04-10

**Authors:** Maximilian Stefani, Wolfgang Mack, Marian Sauter

**Affiliations:** 1 Department of Human Sciences General Psychology, Institute for Psychology, Universität der Bundeswehr München, Werner-Heisenberg-Weg 39, Neubiberg, 85577 Munich, Germany; 2 General Psychology, Institute for Psychology and Education, Ulm University, Ulm, Germany

**Keywords:** Interactive foraging, Haptic search, Attentional constraints

## Abstract

This study investigated how varying difficulty levels modulate haptic foraging performance and search behavior. Thirty-three blindfolded participants had to locate and remove 20 target objects within three conditions – easy feature, conjunction, and hard feature – defined by target-distractor similarity. Response times and target-type switching were measured, and errors were recorded but remained very low across all conditions. Results revealed that higher task difficulty was associated with longer response times. Although participants switched between target types frequently in both the easy and the hard feature conditions, they switched significantly less often in the conjunction condition. This suggests that tasks requiring multiple feature dimensions to distinguish targets from distractors elicit more top-down-driven sequential searching, whereas distinctly defined targets permit more frequent shifts driven by salient cues. Practice effects emerged as participants performed each condition faster when they had already practiced different search conditions. These findings show that haptic search behavior, like visual foraging, is shaped by the interplay of top-down and bottom-up processes. They further highlight that tactile and visual systems may share common representational pathways and that practical considerations, such as target similarity and prior experience, play a crucial role in task efficiency.

## Introduction

In cognitive psychology, foraging is a paradigm that is used to study attentional processes and executive functioning, such as planning. Most of the foraging research focuses on visual foraging, with little attention given to haptic foraging. However, our sense of touch is particularly important in situations where our other sensory impressions do not provide much useful information, for example, when looking for things in our pockets. Research indicates that tactile signals can enhance task performance when vision is limited, often proving more effective than other non-visual signals, for example, kinesthetic signals (Ferber et al., 2009). Moreover, haptic exploration of objects seems to activate brain regions that are typically associated with visual processing, suggesting a substantial overlap in processes between these sensory systems (James et al., 2002).

The vast majority of studies on attention are investigated within the visual domain and discuss the extent to which visual features, such as shape and color (figure-ground organization, e.g., Vecera & O'Reilly, 1998), influence the guidance of our visual processing. This limits the generalizations that can be derived from existing attentional theories. The objective of this study was to gain insight into the mechanisms of selective attention in non-visual, haptic foraging.

### Top-down and bottom-up influences in visual foraging

Visual foraging has its roots in ethnological research, particularly studies on birds. Birds develop specific search patterns to efficiently locate prey, using “searching images” to focus on abundant prey types, which allows them to find food more quickly and effectively (Tinbergen, 1960). Search behavior is influenced heavily by the searcher’s goals (“top-down”) through prior knowledge, expectations, and learned experiences. Top-down processes are crucial in visual foraging because repeated exposure to a stimulus helps create imagined search images, which aid in quickly recognizing and locating targets based on their significance (Kristjánsson, 2006). For example, a person frequently searching for keys in their cluttered home develops a mental image (*target template*) of the keys and their usual locations, which helps focus the search efforts more effectively. In contrast, bottom-up influences are driven by immediate sensory input from the environment, such as a bright red apple standing out against a green background due to its distinct color and shape. Visual attention is thus focused on attributes such as shape, color, and size, which underscores the role of bottom-up processes in guiding search behavior (Wolfe & Horowitz, 2004). Humans, therefore, rely on both top-down and bottom-up processes to guide visual processing.

Even studies on animals show how top-down and bottom-up processes interact. Chickens, for example, use a selective filtering approach in their foraging behavior, focusing strategically on specific types of food rather than searching randomly. This behavior suggests the use of top-down knowledge about the types of food they are seeking, combined with bottom-up cues from their environment (Dawkins, 1971). Similarly, pigeons adjust their search strategies based on the visual distinction between food and its background. When the distinction is clear, pigeons select food randomly. However, as the visual distinction decreases, they focus more on specific types of food, indicating a transition from bottom-up to top-down influences as the task becomes more challenging (Bond, 1983).

A study by Kristjánsson et al. (2014) investigated common attentional constraints in visual foraging in humans. They designed a task where participants were required to locate and touch targets on an iPad screen, with targets varying in their ease of identification. The study featured two conditions: a “feature” condition, where targets were distinguishable by a single attribute, such as color, and a “conjunction” condition, where both color and shape were needed to differentiate targets from distractors. The results revealed a significant difference in foraging behavior between the two conditions. In the feature condition, participants frequently alternated between target categories, leading to multiple short “runs.” As with the pigeons (Bond, 1983), this behavior is indicative of a bottom-up influence, as the distinct features of each target facilitated easy detection, allowing participants to switch between categories without extensive cognitive effort. In contrast, the conjunction condition demanded more focused attention due to the similarity between targets and distractors. Here, participants are typically engaged in exhaustive search patterns within a single category before moving to another, resulting in fewer but longer runs. The authors argue that this shift reflects a stronger reliance on top-down processes, as the participants had to utilize more cognitive resources to identify targets amidst distractors. However, in their study, the increase was to distinguish target objects from quite similar distracting objects, while figure-ground segregation remained easy (uniform black background). In contrast, in the pigeon study, the figure-ground segregation and general clutter of the environment led to increased difficulty – the target objects stayed the same.

### Attentional processes in haptic foraging

Building on the principles of classical visual search, multi-target search examines how individuals locate multiple targets within structured, controlled tasks that emulate real-world search demands. Unlike foraging tasks, multi-target search isolates specific attentional and cognitive mechanisms by imposing constraints such as limited display areas or defined target types. This allows researchers to study how strategies developed in foraging contexts – such as target switching and adaptive behavior – translate into performance in controlled visual search tasks. Searching for multiple targets simultaneously often reduces accuracy compared to single-target searches, particularly when target prevalence is low – a phenomenon termed the “dual-target cost” (Godwin et al., 2010). This effect highlights the cognitive challenges associated with maintaining multiple target representations in working memory. Additionally, target similarity has been shown to significantly mitigate this cost, with better performance when targets share similar features compared to when they are dissimilar (Mestry et al., 2017). Visual working memory can also simultaneously activate multiple target templates, enabling efficient switching between targets during search tasks (Beck et al., 2012). This capability is particularly relevant in foraging tasks that require identifying multiple target types amidst distractions. These findings suggest that attentional control operates through a dynamic interaction between multiple active representations, facilitating flexible context-dependent search strategies.

However, while the shape of objects, i.e., their physical properties, can be perceived very quickly in a visual search, where color or certain shapes are sufficient, this is not possible in a purely tactile search. No three-dimensional (3D) features can be easily identified. Only their two-dimensional (2D) shape can be recognized within a few milliseconds (Lederman & Klatzky, 1997). If a 3D object has to be recognized and thus spatially processed, the process is very slow and error-prone (Klatzky et al., 1993). Thus, physical exploration strategies play a decisive role in haptic search. Systematic search strategies, such as parallel swings and spirals, are more common in one-finger searches, indicating a methodical approach to haptic exploration (Morash, 2014). Furthermore, haptic search strategies appear to be guided by prior knowledge and thus facilitate the haptic classification process. For example, the participants had to recognize whether it was a spoon or a fork.

To explain this search behavior, Lederman and Klatzky (1987) developed a concept of Exploratory Procedures (EPs). EPs are stereotypical movement patterns, each tailored to extract specific object-related variables; for example, lateral movements are used to assess surface texture, pressure is applied to determine the hardness, and constant touch is used for temperature. Lederman and Klatzky (1993) describe a two-stage process in haptic exploration using different EPs. In the first stage, basic EPs collect immediate tactile input (bottom-up) about properties like size and rough shape. The second stage involves more specialized, time-consuming EPs for detailed properties like precise shape. This stage integrates more top-down processes, where the brain uses prior knowledge, expectations, and cognitive strategies to guide exploration and interpret sensory data. The choice of EPs itself can be influenced by top-down factors such as task requirements. Thus, when participants had to differentiate between a spoon and a fork, they engaged in a self-terminating search, utilizing fundamental EPs until they could dismiss the erroneous identification without extensively employing specialized second-stage EPs (Lederman & Klatzky, 1990).

Recent studies have expanded on this framework, showing that EPs facilitate object recognition by focusing on material versus geometric properties and integrating these dimensions. For instance, lateral motion helps discern roughness, while pressure aids in estimating compliance (Jansen et al., 2013). This adaptability in EPs illustrates how haptic foraging strategies are influenced by both the physical characteristics of objects and the prior knowledge of the explorer. Also, in robotics, the implementation of EPs in artificial hands reflects the capabilities of the human sense of touch. Robotic systems use specific tactile and kinesthetic data to recognize and manipulate objects, mirroring human haptic exploration strategies (Stansfield, 1992).

Overall, like visual foraging, haptic foraging involves an integration of top-down and bottom-up influences similar to visual foraging, integrating prior knowledge, expectations, and immediate sensory input to optimize the search and recognition processes. However, the overall exploration of the environment is fundamentally different.

### Interactive visual-haptic foraging

The tactile system lacks the same level of global spatial resolution as the visual system. While vision can process numerous details simultaneously across a broad field, tactile perception relies on sequential exploration to acquire information. This distinction suggests that the attentional mechanisms and strategies governing haptic foraging may differ substantially from those in visual foraging. People are highly proficient at identifying objects with their bare hands while blindfolded, achieving almost 100% accuracy within just 2–3 s per object, demonstrating that identification by touch alone is entirely feasible (Klatzky et al., 1985). However, in mixed visual-haptic settings, humans tend to rely more on their eyes than their hands for searching (Fragaszy et al., 2010). Differences in search strategies have been observed between capuchin monkeys and humans, with monkeys favoring manual searches for sunflower seeds, while humans primarily use visual strategies and are less likely to employ their hands. For haptic search, two distinct phases have been identified: an initial broad exploration phase, where all fingers are engaged in locating targets and rely heavily on bottom-up sensory input, and a subsequent detailed examination phase, primarily involving the middle and index fingers (Metzger et al., 2019).

Brain-imaging studies have shown that prior haptic experience with novel objects leads to enhanced activation in visual areas when these objects are subsequently viewed (James et al., 2002). This finding suggests that haptic exploration activates both somatosensory and visual areas in the brain, indicating that the same object-representation systems used in visual perception are also engaged during haptic tasks (Chow et al., 2022). The activation of visual areas during haptic tasks shows how prior visual experiences and expectations enhance the interpretation of tactile information. This shared processing pathway between sensory modalities demonstrates the brain’s remarkable ability to integrate information from multiple sources to create a cohesive perceptual experience (Chow et al., 2022). Furthermore, the effectiveness of visuo-haptic object-selective brain regions is enhanced with increasing stimulus salience. The neuronal convergence of visual and haptic inputs for processing object shapes indicates that the brain’s sensory processing systems are highly adaptable and capable of integrating and prioritizing inputs based on their salience and the task at hand (Lacey & Sathian, 2014). This flexibility allows the brain to optimize its processing resources and improve overall perceptual performance. Thus, haptic exploration activates both somatosensory and visual areas in the brain, suggesting that the same object-representation systems used in visual perception are also engaged during haptic tasks. This indicates a shared processing pathway between these sensory modalities (James et al., 2002). There have been few studies that investigated a joint visual-haptic foraging that was phrased as an interactive search to differentiate it from a screen-based search or foraging. A study by Sauter et al. (2020) introduced an interactive foraging paradigm using LEGO® bricks to bridge the gap between traditional computerized visual search and real-world foraging behaviors. By arranging simple LEGO® bricks on tabletop trays, the researchers aimed to ensure comparability with classical computerized visual foraging studies while providing room for easily increasing the complexity of the search environment. Their findings indicated that targets were grasped more slowly when more distractors were present, and there were significant differences between various difficulty conditions, aligning with classical visual search research and revealing similarities to research in natural scenes. Hout et al. (2022) expanded the use of LEGO® bricks to investigate active and passive strategy use in interactive foraging tasks. Their methodology involved participants searching for specific LEGO® targets within cluttered trays, yielding patterns of diminishing returns in response times as targets were removed. Importantly, they demonstrated that active search strategies – characterized by deliberate physical interaction and directed attention – were more effective than passive strategies, where participants relied on incidental encounters with targets.

## Rationale

This study investigated the impact of difficulty levels on search behavior in haptic foraging tasks, specifically focusing on the interaction between top-down and bottom-up influences. By manipulating the similarity between targets and distractors, we aimed to understand how these factors shape search strategies and overall task performance in the haptic domain.

Previous studies (Kristjánsson et al., 2014; Sauter et al., 2020) have shown that difficulty levels – defined by the similarity between targets and distractors – significantly influence search behavior. In their study, participants’ performance varied across conditions, with easier tasks (distinct targets and distractors) leading to shorter search times and frequent category switching, indicative of bottom-up processing. In contrast, more challenging tasks (similar targets and distractors) required greater reliance on top-down processes, as participants tended to focus on one category at a time, resulting in longer search times and fewer switches.

In this study, we extend these findings to haptic foraging by examining how different levels of difficulty influence search behavior and strategy selection. The difficulty conditions are defined as follows: (1) Easy Feature Search: Targets are easily distinguishable from distractors by a single tactile feature. (2) Hard Feature Search: Targets and distractors are quite similar but can be differentiated on a single feature dimension. (3) Conjunction Search: Targets are defined by a conjunction of two features, making them harder to distinguish from distractors. Our study uses different LEGO® bricks to create these search conditions. The bricks can be differentiated by two features, which we refer to as *shape* and *structural configuration*. Shape refers to the overall geometry of the bricks, such as whether they are rectangular (e.g., Brick 2 × 2 and Brick 2 × 3) or cylindrical (e.g., Round Brick 1 × 1 and Nose Cone Small 1 × 1). Structural configuration pertains to the arrangement of studded surfaces, including the number and layout of studs on the top (e.g., 2 × 2 vs. 2 × 3 bricks) or the presence of unique design elements (e.g., equal vs. unequal surface size on top vs. bottom).

We anticipate that participants will complete the Easy Feature Search condition more quickly than both the Hard Feature and the Conjunction Search conditions, with the Hard Feature condition taking the longest. This prediction is consistent with visual foraging research (e.g., Kristjánsson et al., 2014; Sauter et al., 2020), where low target–distractor similarity facilitates faster detection. However, because tactile foraging often requires sequential, hands-on exploration (Lederman & Klatzky, 1987, 1993), it remains unclear whether the speed advantage observed in simpler visual tasks will directly translate to haptic contexts.

We further predict that participants will exhibit more frequent switching between target types in the Easy Feature condition than in the Hard Feature or Conjunction conditions. In visual search, highly salient features tend to “pop out,” encouraging rapid alternation between multiple target categories (Kristjánsson et al., 2014). In contrast, purely tactile exploration may not afford such immediate perceptual cues, potentially compelling individuals to focus on one category for longer. Comparing switching behavior across these conditions will clarify whether the frequent switching observed in visual foraging also characterizes tactile tasks.

Beyond these hypotheses, an important question is whether the unique demands of haptic perception moderate the impact of task difficulty. Haptic exploration depends on sequential manual contact and can involve detailed exploratory procedures (EPs) to extract specific object properties (Lederman & Klatzky, 1987). Even in conditions designed to be “easy,” participants may engage in extended tactile inspection if immediate bottom-up cues (e.g., a distinct texture or shape) are insufficient to confirm an object’s identity (Morash, 2014). This dynamic interplay between bottom-up signals and top-down factors (e.g., searching “images” built on prior knowledge; Lederman & Klatzky, 1993; Tinbergen, 1960) may yield differences in search performance that are not fully predicted by visual foraging models.

Finally, it remains an open question whether increases in target–distractor similarity will enhance reliance on top-down processes during haptic search. In visual contexts, difficult searches typically push participants to adopt methodical or exhaustive verification strategies (Kristjánsson et al., 2014). However, because tactile exploration is inherently serial – often requiring multiple EPs (e.g., pressing, lateral movements) to distinguish subtle physical features (Lederman & Klatzky, 1990) – these strategies may be even more pronounced in haptic foraging. By examining how participants adapt to progressively challenging tactile conditions, we can determine if the characteristic patterns of visual foraging (e.g., fewer switches, thorough exploration within a single category) also emerge when vision is excluded, thus refining our understanding of cross-modal attention and search behavior (Chow et al., 2022; James et al., 2002).

## Method

### Participants

The study was conducted with a total of 33 blindfolded participants, comprising 18 males and 15 females. The participants were primarily students from the University of the Bundeswehr Munich (N = 25) and civilian students from various other institutions (N = 8). The average age of the adjusted sample was 23.03 years (range: 20–28 years), and four were left-handed. The experience levels with bricks among participants varied, with 14 participants having little to no experience and 19 participants possessing a high degree of experience. Three participants had to be excluded due to technical difficulties; they were not included in the sample description.

All participants provided informed consent and were given course credit per hour attended as compensation for their participation if this was requested. All procedures performed in this study were in accordance with the 1964 Helsinki Declaration and were typically declared exempt from needing a full vote by the local ethics committee. We based our sample size on a power analysis informed by effect sizes reported in comparable interactive foraging paradigms. In Experiment 2 of Sauter et al. (2020), the main effect of search type on response times was very large (*η*^*2*^_*p*_ = 0.77), whereas specific pairwise contrasts ranged from medium to large (Cohen’s d_z_ of 0.42, 1.05, and 1.43). To be conservative – particularly since one effect size (color vs. conjunction) was more modest – we used G*Power 3.1 to estimate the required N for a repeated-measures ANOVA (three within-subject conditions) targeting a medium effect size of *f* = 0.25 (roughly in line with *d*_*z*_ ≈ 0.42), *α* = 0.05, *β* = 0.20 (80% power), and an assumed correlation among measures of 0.50. This calculation suggested approximately 28 participants. We increased that to 36 to account for possible attrition or data exclusions, thereby ensuring adequate power to detect medium-to-large effects.

### Setup

**Fig. 1:**
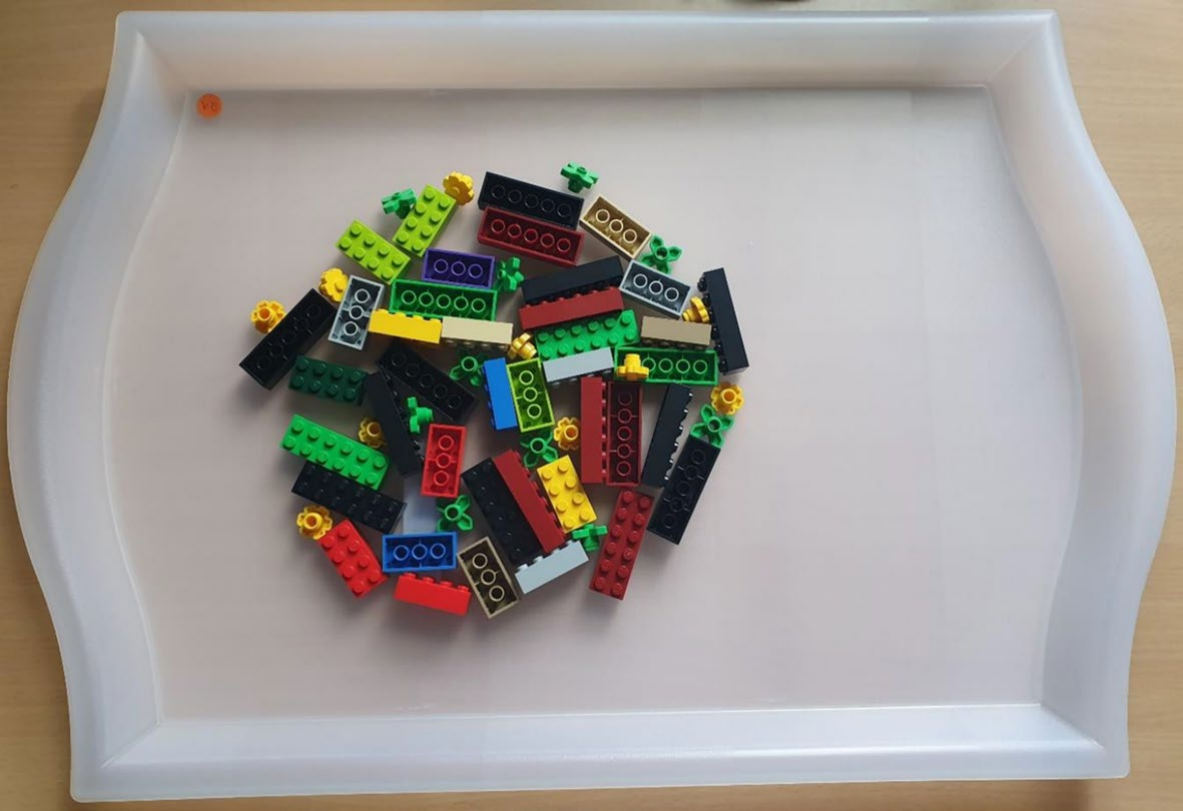
Search display for an easy feature search condition

OpenSesame (Mathôt et al., 2012), which was operated by the test administrator, was used to measure response times, switching operations, and error rates. Each search target and each distractor were assigned to a button that was pressed when the participant placed a brick next to the tablet. There were no instructions on how to search, i.e., it was possible to search with one or both hands. The bricks were commercially available LEGO® bricks, which were distributed on a tablet (48 cm × 35 cm) (see Fig. 1). The tablet served as a search display and was positioned in front of the participant.

### Task

Each search display contained 60 bricks consisting of four different types. Two types were distractors with 20 bricks each, and two types were targets with 10 bricks each. See Table 1 for a short overview, and all brick sets are in Tables 4, 5 and 6 in the Appendix. The arrangement of the search display differed in the haptic search conditions easy feature, conjunction, and hard feature. If the distractors are markedly different from the targets (e.g., different number of studs), an easy feature haptic search was assumed. The search condition was therefore characterized by a low target–distractor similarity but a high target–target and distractor–distractor similarity. In the conjunction search condition, the same bricks were used as in the easy feature search condition, but one distractor and target were swapped, resulting in a high target–distractor similarity but a low target–target and distractor–distractor similarity. In the hard feature search condition, highly similar bricks were used, resulting in a high target–distractor similarity, as well as a high target–target and distractor–distractor similarity. The experimenter ensured that no large clusters of identical bricks were formed, manually mixing them if necessary.

**Table 1:**
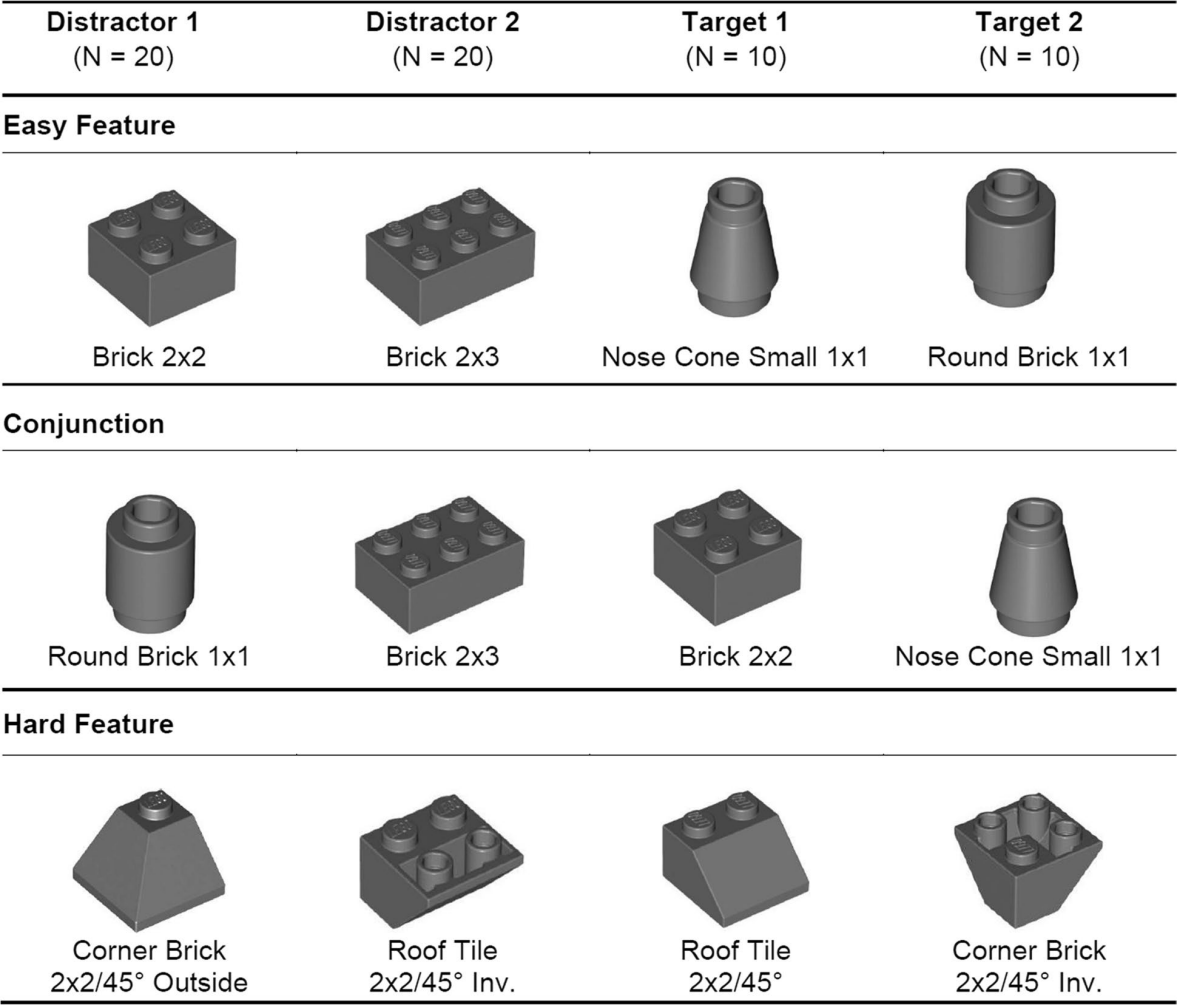
Configuration example for the condition of the different sets

### Design

Since this experiment was a purely haptic search, the participants’ eyes were covered with a sleep mask after they had 30 s to memorize the two search targets (visual and haptic). Participants were instructed that they had to find 20 targets in each haptic search trial. Each target was to be found as quickly as possible and, once found, placed to the left of the tray for the experimenter to collect and record. This procedure was applied to all bricks placed by the participant, irrespective of whether they were designated as targets or distractors. Where a distractor brick was placed, the experimenter refrained from offering a comment. The intervention was only undertaken in instances where it was evident that the participant had erroneously identified a distractor as a target and was actively searching for it. The placement of three consecutive distractors served as the basis for this assumption. The haptic search was automatically stopped when all 20 targets were found.

All haptic search conditions were carried out five times with different brick sets, resulting in a total of 15 haptic searches. A haptic search condition was always run through completely, and each participant performed the different brick sets within a condition in the same order. However, there were two groups, with one group assigned the haptic search sequence easy feature – hard feature – conjunction and the other conjunction – hard feature – easy feature.

At the end of each haptic search condition, the participants completed a short questionnaire on their haptic search behavior. They were asked to report whether they had searched with their left, right, or both hands, and to rank the five searches from easiest to most difficult, describing the exact search strategy (free text), and rating the search difficulty from very easy (1) to very difficult (7).

### Analyses

RStudio 2023.06.1 (RStudio Team, 2016) and R 4.3.1 were used with the tidyverse package version 1.3.0 (Wickham, 2017) for data preparation and creating figures and JASP version 0.18.3 to calculate repeated-measures ANOVAs and post hoc analyses (JASP Team, 2023). We calculated median response times and error rates per participant and condition if not stated otherwise. Post hoc tests were conducted on the estimated marginal means with Bonferroni’s t-test, and we used the Bonferroni equation for computing degrees of freedom.

### Transparency and openness

We report how we determined our sample size, all data exclusions (if any), all manipulations, and all measures in the study. All data, analysis code, and research materials are available on the Open Science Framework at https://osf.io/pcgxa/. This study’s design and its analysis were not pre-registered.

## Results

### Error rates

The error rate was calculated as the proportion of distractors among the total number of bricks identified under a specific haptic search condition. In the easy feature condition, no errors were made, indicating that none of the distractors were mistakenly identified as targets. The mean error rate for the conjunction condition was less than 1%. In the hard feature condition, the average error rate was also less than 1%. However, on closer examination, the highest error rate of 2.9% was found in the first search of the hard feature condition. Due to the very low error rates, we refrained from further analysis.

### Response times

**Fig. 2:**
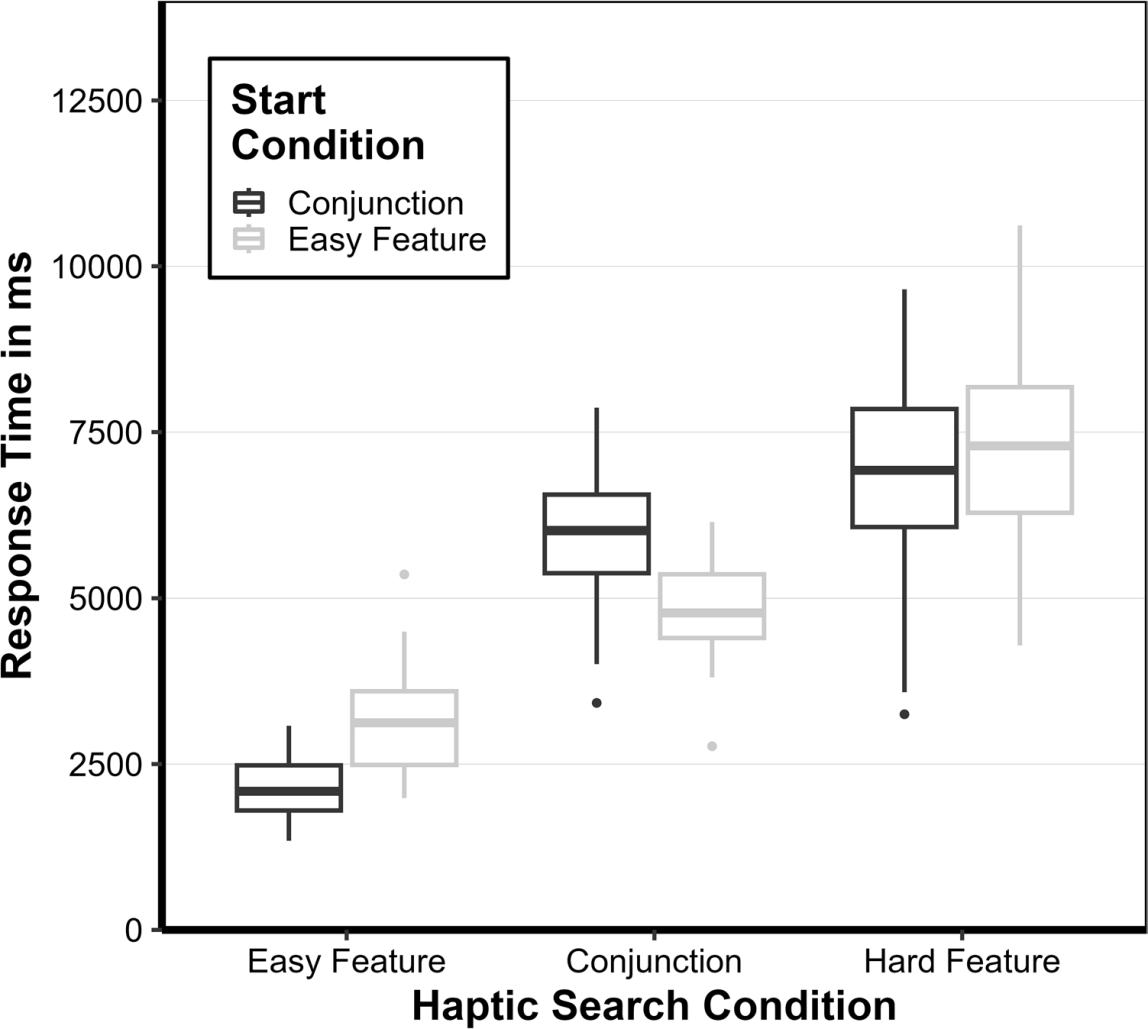
Median response times for the haptic search conditions easy feature, conjunction, and hard feature depending on start condition (easy feature vs. conjunction). The lower and upper hinges (boxes) correspond to the first and third quartiles (the 25 th and 75 th percentiles). Data beyond the end of the whiskers are outliers (black dots)

To describe the differences between the haptic search conditions as well as the differences in the start condition (easy feature vs. conjunction haptic search), a repeated-measures ANOVA with the within-subject factors haptic search condition (easy feature, conjunction, hard feature) and between-subject factor start condition (easy feature or conjunction). The main effect of the haptic search condition, *F(*2, 62) = 176.14, *p* < 0.001, *η*^*2*^_*p*_ = 0.684, was significant but not the main effect for the start condition, *F(*1, 31) = 0.14, *p* = 0.712, *η*^*2*^_*p*_ = 0. 003. The interaction of the haptic search condition and start condition, *F(*2, 62) = 10.44, *p* < 0.001, *η*^*2*^_*p*_ = 0.114, was significant. Participants located the bricks in the easy feature search *M*_*diff*_ = 2,653 ms, 95% CI [2,088; 3,217], faster than in the conjunction search, t(31) = 11.56, *p* < 001, *d*_cohen_ = 2.153, and in the conjunction search *M*_*diff*_ = 1,611 ms, 95% CI [1,047; 2,176], faster than in the hard feature search, *t*(31) = 7.02, *p* < 001, *d*_cohen_ = 1.308 (see also Fig. 2). The condition that was started with, however, was noticeable in both start conditions. Participants were faster, *M*_*diff*_ = 1,072 ms, 95% CI [− 233; 2,378], if they had completed this condition following the two other haptic search conditions, *t*(31) = 2.50, *p* = 0.045, *d*_cohen_ = 0.870. The same applied to the conjunction haptic search condition. If the condition was the last, the participants were faster than when they started with it, *M*_*diff*_ = 1001 ms, 95% CI [− 305; 2,307], *t*(31) = 2.33, *p* = 0.045, *d*_cohen_ = 0.812. The hard feature haptic search condition was not affected by the start condition, *M*_*diff*_ = 307 ms, 95% CI [− 999; 1,313], *t*(31) = 0.72, *p* = 0.477, *d*_cohen_ = 0.249.

This demonstrated that the more challenging the haptic search condition was, the more time was needed to find all 20 targets. Whilst the haptic search that was started mattered, it did not change the effects between the haptic search conditions, and rather showed a practice effect.

### Switching rate

**Fig. 3:**
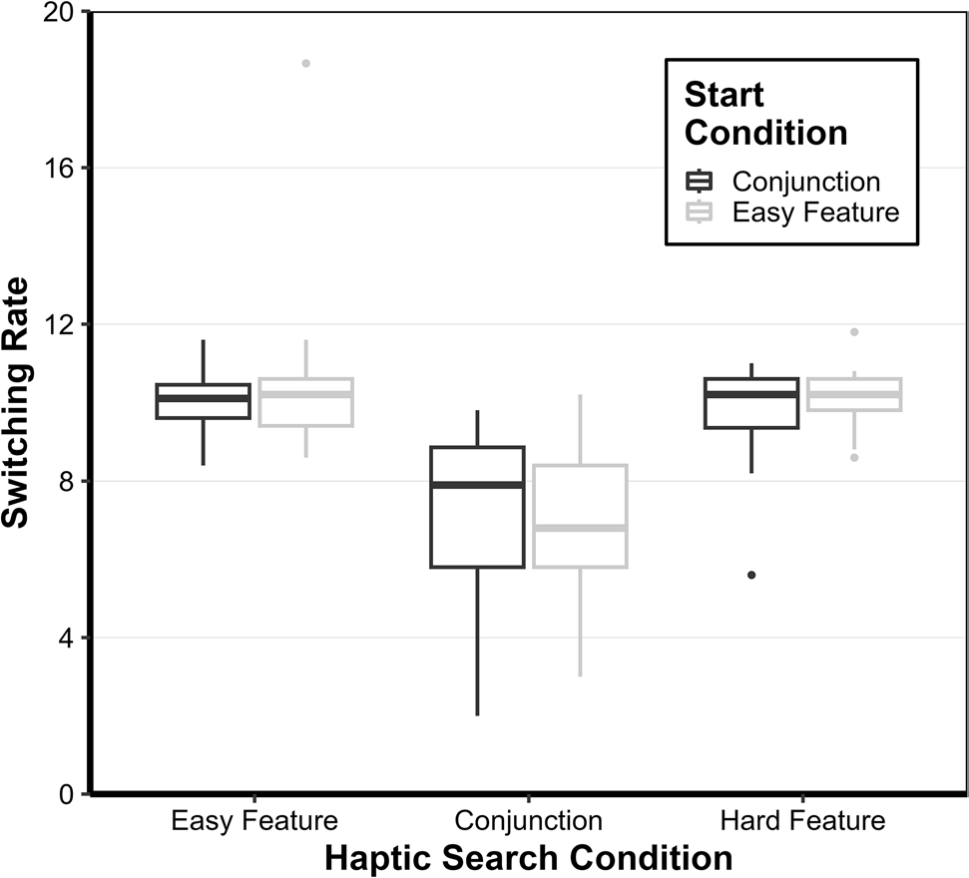
Switching rate for the haptic search conditions easy feature, conjunction, and hard feature depending on start condition (easy feature vs. conjunction). The lower and upper hinges (boxes) correspond to the first and third quartiles (the 25 th and 75 th percentiles). Data beyond the end of the whiskers are outliers (black dots)

The switching rate – in other words, how often the search was switched between the two search targets, was also of interest. Therefore, a repeated-measures ANOVA with the within-subject factors haptic search condition (easy feature, conjunction, hard feature) and between-subject factor start condition (easy feature or conjunction) was conducted. The main effect of the haptic search condition, *F(*2, 62) = 94.23, *p* < 0.001, *η*^*2*^_*p*_ = 0.430, was significant but not the main effect for the start condition, *F(*1, 31) = 0.36, *p* = 0.554, *η*^*2*^_*p*_ = 0. 005. The interaction of the haptic search condition and start condition, *F(*2, 62) = 0.69, *p* < 0.505, *η*^*2*^_*p*_ = 0.012, was also not significant (see Fig. 3). No differences were observed between the easy feature and hard feature haptic search, *M*_*diff*_ = 0.4, 95% CI [− 0.5; 1.3], *t*(31) = 0.985, *p* = 0.328, *d*_cohen_ = 0.221. In contrast, lower switching rates were observed for conjunction search compared to both easy feature search, *M*_*diff*_ = 3.1, 95% CI [2.2, 4.0], *t*(31) = 8.41, *p* < 0.001, *d*_cohen_ = 1.888, and hard feature haptic search, *M*_*diff*_ = 2.7, 95% CI [1.8, 3.6], *t*(31) = 7.42, *p* < 0.001, *d*_cohen_ = 1.667.

Consequently, participants switched the search target substantially less in the conjunction haptic search, which means that they apparently tried to find one search target first and then the other.

### Additional analysis

In order to complement the main findings, a series of exploratory analyses were conducted to further investigate various aspects of human haptic search behavior. Below, we briefly summarize the key observations.

The first set of analyses addressed whether there was a relationship between target-type switching and search or response time. Correlations were examined separately for the easy feature, hard feature, and conjunction conditions. None of them were significant.

Participants were also asked which aspect of the experiment they found most difficult. Their free-text responses were categorized into five main themes: the high target–distractor similarity in the hard feature condition, maintaining order on the tablet, memorizing target features, handling new or very small pieces, and miscellaneous single mentions. The majority (18 participants) indicated that the hard feature condition’s high similarity was the most demanding element. Four participants each cited tablet arrangement, memorizing targets, and handling small or unfamiliar pieces, whereas three participants either provided no information or gave unique responses (e.g., limited encoding time). These descriptive results align with the main analyses, where the hard feature condition was objectively the most time-consuming and subjectively most challenging.

A related question was whether the amount or type of visual input before each trial influenced haptic performance. Participants were free to use the entire 30-s encoding interval or begin searching earlier; on average, they used about 18 s for easy feature, 21 s for conjunction, and 24 s for hard feature haptic searches. A positive correlation emerged between this “encoding time” and response time in the easy feature condition (*r* = 0.404, *p* = 0.020), suggesting that participants who studied the bricks more extensively before blindfolding were somewhat slower to respond once they began searching. By contrast, no significant correlations were observed for the conjunction and hard feature conditions (*p* > 0.17). Furthermore, some participants spontaneously closed their eyes during the encoding phase, while others kept them open. Those who kept their eyes open throughout tended to have faster response times, *t*(31) = − 3.041, *p* = 0.005, *d* = 1.071. Notably, the number of target-type switches did not differ as a function of this behavior.

Finally, participants’ self-reported experience with LEGO® bricks showed no meaningful correlation with the total time required to complete the tasks (*r* = − 0.156, *p* = 0.385). In line with the main results, subjective difficulty ratings confirmed that the easy feature was perceived as the least challenging and the hard feature as the most challenging haptic search. Although participants who rated the tasks as more difficult sometimes reported taking longer in easy feature or hard feature conditions, these small correlations (*r* values around 0.20–0.25) were not statistically reliable.

## Discussion

The present study investigated how varying difficulty levels – operationalized via target–distractor similarity – impact haptic foraging performance. Overall, three primary results emerged. First, target-type switching was highest in the easy feature and hard feature conditions, whereas it was comparatively lower in the conjunction condition. This suggests that in haptic foraging, as in visual foraging (Kristjánsson et al., 2014), distinct or highly salient tactile features encourage frequent alternation between target types. Conversely, if targets have to be identified by combining several characteristics, the participants apparently tend to focus on one target type at a time.

Second, search efficiency in terms of response times worsened as the tasks became more difficult. The easy feature haptic searches (single salient feature) were completed most rapidly, the conjunction haptic searches required moderate search times, and the hard feature haptic searches showed the longest search times. These results align with well-established findings in visual search literature, where increases in target–distractor similarity compel more exhaustive search strategies (Treisman & Gormican, 1988; Wolfe et al., 1989). Yet, the unexpected observation that switching rose again for the hard feature condition indicates a more complex interaction of bottom-up and top-down factors than a simple continuum from easy feature to hard feature haptic searches.

Third, error rates remained very low across all conditions, indicating that participants were generally successful in tactile identification despite the absence of vision. This points to the effectiveness of haptic exploratory procedures (Lederman & Klatzky, 1987) and also underscores the potential influence of prior knowledge – particularly for participants familiar with LEGO® bricks – on maintaining accuracy.

### Bottom-up and top-down interplay under increasing task demands

A central takeaway from our study is the dynamic balance of bottom-up and top-down processes that become apparent as haptic search tasks become more challenging. In the easy feature haptic searches, distinct physical features (e.g., shape or texture) can directly “pop out” to the sense of touch, resembling the bottom-up salience effects seen in visual foraging (Treisman & Gormican, 1988). In these searches, participants not only locate targets more quickly but also switch between target types at higher rates.

As target–distractor similarity increases, however, the benefits of bottom-up salience diminish. Participants must rely on top-down processes – maintaining explicit mental representations of two or more target templates and sequentially confirming each object’s identity through tactile exploration (Lederman & Klatzky, 1993; Wolfe et al., 1989). The resultant slowing of response times and reduced switching (particularly in the conjunction condition) reflect this more deliberate strategy. Interestingly, the hard feature condition showed both slowed search and increased switching, suggesting that participants repeatedly verified objects across target categories in an effort to resolve ambiguity – similar to “double-checking” or “reset” strategies in challenging visual searches (Kristjánsson et al., 2014).

One unexpected or at least less intuitive result is the increased switching rate in the hard feature condition. Based on visual foraging findings, the initial assumption might have been that extreme similarity would lead participants to methodically finish one target category before moving on to the next, yielding minimal switching. Instead, our data show that participants continued to alternate between target types. This pattern may indicate: (1) Ongoing uncertainty resolution: When objects are highly similar, participants might identify a possible target, second-guess it, and then shift to the other category to verify if that category’s features are easier to detect. (2) A serial “back-and-forth” strategy: Since participants knew there were two target types, it may have been more efficient in certain instances to move momentarily to the other target definition in working memory, especially if the initial impression was inconclusive. Such findings underscore how task demands can prompt more complex, iterative attentional strategies rather than a simple one-category-at-a-time approach. In this sense, “extreme” difficulty does not merely force an exhaustive search within one category but can also drive frequent toggling between multiple incomplete mental templates. Taken together, under high similarity haptic foraging prioritizes a hybrid search mode, similarly influenced by both structured (top-down) verification and opportunistic (bottom-up) tactile cues.

### Relation to the broader literature

Our findings highlight how *exploratory procedures* (EPs) – the systematic tactile movements described by Lederman and Klatzky (1987) – contribute to performance under varying target–distractor similarities. In the easy feature condition, participants likely employed broad, rapid EPs (e.g., global shape checks) that facilitated frequent target-type switching based on highly salient tactile cues. By contrast, the longer search times and fewer switches observed in the conjunction condition suggest a reliance on more specialized EPs (e.g., contour following), which are slower and cognitively more demanding (Klatzky et al., 1993). Interestingly, the hard feature condition demonstrated an unexpected increase in switching, implying that participants shifted between broad and specialized EPs to resolve high ambiguity.

A key insight from multi-target search research is that *category-based representations* substantially influence how efficiently participants switch among different targets (Beck et al., 2012). In visual search, when targets share a single defining category (e.g., color), observers can easily alternate between them; however, conjunction or multiple-category targets impose a higher cognitive load (Menneer et al., 2019). Our data suggest that similar mechanisms apply in haptic search. For instance, when participants perceived the targets as belonging to distinct tactile “categories” (i.e., obviously different shapes or stud patterns), they switched more freely. Conversely, when discriminating targets fell into more overlapping or complex categories (as in the conjunction and hard feature conditions), participants were more methodical, which likely limited the rate of switching (Godwin et al., 2010; Wolfe et al., 2019).

The serial nature of haptic exploration also places unique demands on working memory. While visual foraging can exploit initial switches of parallel processing, haptic tasks require continuous item-by-item checks, thereby heightening cognitive load. Research on visual multi-target search indicates that maintaining multiple search templates in memory can reduce accuracy and slow response times (Godwin et al., 2010; Wolfe et al., 2019). These constraints may be amplified in haptic foraging, where, for instance, hard feature tasks force participants to juggle multiple subtle properties (shape, texture, stud configuration) within a limited-capacity system. Indeed, our data suggest that longer encoding times – during which participants visually and tactually studied the target bricks before blindfolding – did not lead to faster search; instead, those who spent more time encoding objects tended to record slower response times. This outcome implies that elaborate or extended initial encoding may raise the overall cognitive load associated with storing and comparing target templates, which makes subsequent haptic identification more difficult rather than faster. Thus, tactile contact must be interpreted, compared to mental templates, and either rejected or confirmed – an especially resource-intensive process as task difficulty increases.

### Methodological and practical Implications

By using physical LEGO® bricks rather than computer-generated stimuli, we more closely approximate the tactile conditions encountered in everyday scenarios – such as searching a cluttered drawer or bag without visual cues. This approach helps bridge the gap between laboratory-based attention studies and real-world search behaviors, demonstrating that the complexity of real tactile interactions can mirror and extend findings from the visual domain.

Insights into exploratory procedures (EPs) and the interplay of top-down and bottom-up processes have valuable implications for training. For instance, programs designed for individuals with visual impairments could emphasize efficient category-based strategies and working memory management, helping people learn to optimize tactile exploration when vision is compromised. Similarly, occupational therapists may employ structured tasks with systematically varying target–distractor similarity to gradually build haptic discrimination skills.

Given the increasing reliance on tactile feedback in consumer and industrial technologies (e.g., smartphone vibrations, haptic virtual reality (VR) controllers), understanding how people discriminate objects by touch can inform the design of more intuitive interfaces. Tailoring haptic cues to reduce target–distractor confusion – especially under conditions where precise or rapid tactile identification is necessary – could enhance user performance and reduce cognitive load in professional settings (e.g., surgery, robotics, or assembly lines).

Finally, our findings on target switching and search efficiency show that attentional constraints observed in visual search paradigms also apply to touch. By systematically comparing how each modality manages difficulty and cognitive load, future work can develop comprehensive theories of attention that span multiple sensory domains and real-life contexts.

## Conclusion

This study demonstrates that haptic foraging, much like its visual counterpart, is governed by a dynamic interplay of bottom-up and top-down processes, with task difficulty and target–distractor similarity significantly shaping haptic search strategies. Participants showed more frequent switching when targets were clearly distinct (easy feature) or extremely similar (hard feature), whereas they employed more focused, less frequent switching under moderate similarity (conjunction). By adopting a real-world, three-dimensional approach, the findings extend our understanding of attention beyond visual paradigms and underscore the importance of considering multisensory mechanisms, even when vision is excluded.

## Figures and Tables

**Table 4:**
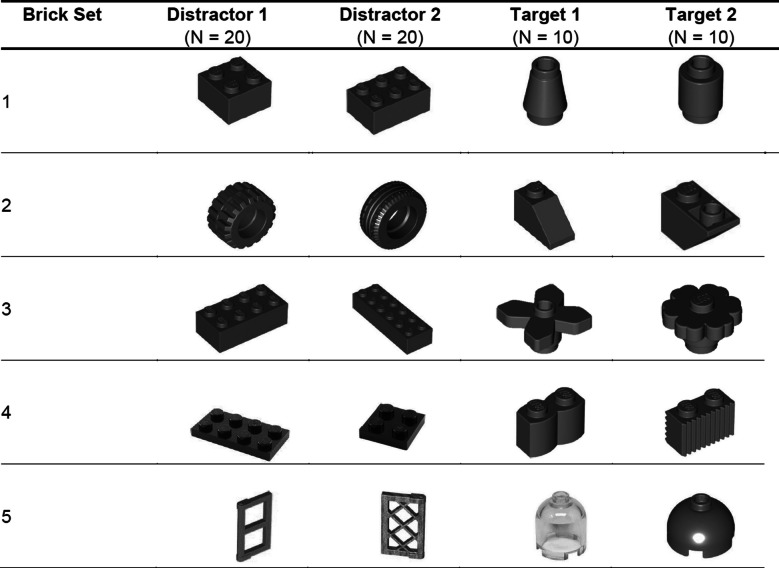
Configurations of all brick sets for the easy feature haptic search condition

**Table 5:**
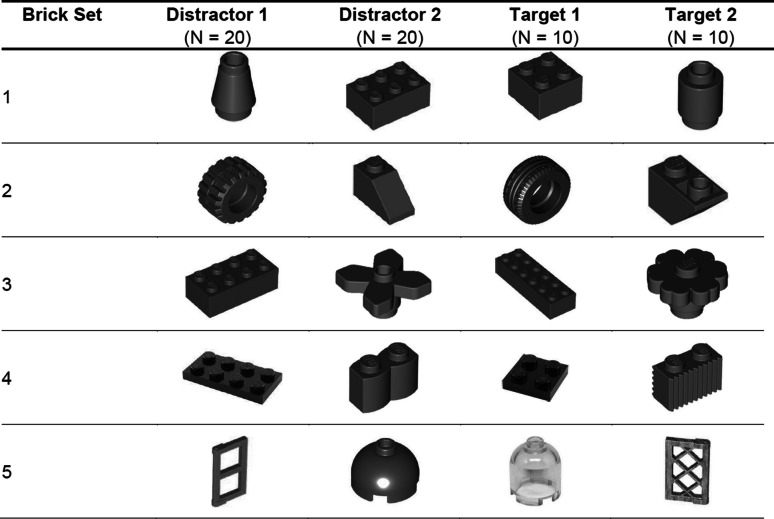
Configurations of all brick sets for the conjunction haptic search condition

**Table 6:**
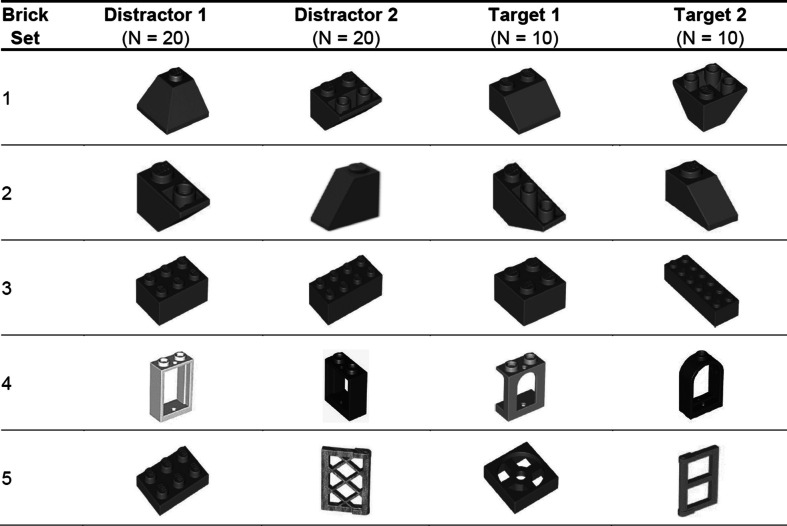
Configurations of all brick sets for the hard feature haptic search condition

## Data Availability

The datasets, material, and code generated and/or analyzed during the current study are available via the Open Science Framework at: https://osf.io/pcgxa/. This study was not pre-registered.

## Appendix

### Differences between the brick sets

Since different brick sets were used in each haptic search condition, a repeated-measures ANOVA with the within-subject factors haptic search condition (easy feature, conjunction, hard feature) and brick set (one to five), were conducted. The aim was to demonstrate whether the selected bricks matched the intended conditions and were comparable within them. The main effects of haptic search condition, *F(*2, 62) = 130.42, *p* <.001, *η*^*2*^_*p*_ = 0.474, were significant. The brick sets insert therefore generally contain the condition we have assumed (further analyses in the section *Response times*). The interaction haptic search condition and brick set, *F(*8, 248) = 16.45, *p* <.001, *η*^*2*^_*p*_ = 0.157, was also significant. Only minor, albeit partially significant, differences were found in the easy feature and conjunction search (see Table 2). However, the first brick set as well as the first search in the hard feature search is particularly noticeable, as the response time is significantly slower than all other brick sets.

**Table 2:** Median response times for each brick set in each haptic search condition (easy feature, conjunction, hard feature) in milliseconds with standard deviation in brackets

	Brick set									
Haptic search condition	*1*		*2*		*3*		*4*		*5*	
Easy feature	3,458	(1,673)	2,149	(905)	2,617	(1,195)	3,370	(1,313)	1,925	(783)
Conjunction	5,784	(1,636)	5,569	(2,044)	6,311	(2,156)	4,833	(1,167)	4,174	(1,269)
Hard feature	10,439	(4,171)	7,160	(2,162)	5,873	(1,551)	6,472	(2,105)	5,018	(1,674)

The same analysis was performed for the switch rate. The main effects of haptic search condition, *F(*2, 62) = 53.21, *p* <.001, *η*^*2*^_*p*_ = 0.215, were significant. The brick sets insert therefore generally contain the condition we have assumed (further analyses in the section *Switching rate*). The interaction haptic search condition and brick set, *F(*8, 248) = 11.99, *p* <.001, *η*^*2*^_*p*_ = 0.155, was also significant. No differences were observed in the haptic search for easy and hard features (see Table 3). Nevertheless, there were large differences in conjunction searches: the participants only switched 3.8 times for brick set 2 and more than twice as often for brick set 4 (10.3 switches) Tables 4, 5 and 6.

**Table 3:** Switching rate for each brick set in each haptic search condition (easy feature, conjunction, hard feature) in milliseconds and standard deviation in brackets

	Brick set									
Haptic search condition	*1*		*2*		*3*		*4*		*5*	
Easy feature	10.2	(1.5)	10.4	(1.6)	10.0	(2.0)	10.5	(1.5)	9.0	(2.4)
Conjunction	7.7	(3.3)	3.9	(3.4)	7.1	(3.5)	10.3	(2.6)	6.9	(4.3)
Hard feature	9.3	(2.3)	10.4	(2.3)	9.9	(2.0)	9.7	(2.0)	10.3	(1.6)
